# Genetic Evidence for a Mitochondriate Ancestry in the ‘Amitochondriate’ Flagellate *Trimastix pyriformis*

**DOI:** 10.1371/journal.pone.0001383

**Published:** 2008-01-02

**Authors:** Vladimir Hampl, Jeffrey D. Silberman, Alexandra Stechmann, Sara Diaz-Triviño, Patricia J. Johnson, Andrew J. Roger

**Affiliations:** 1 Department of Biochemistry and Molecular Biology, Dalhousie University, Halifax, Nova Scotia, Canada; 2 Department of Biological Sciences, University of Arkansas, Fayetteville, Arkansas, United States of America; 3 Department of Microbiology, Immunology, and Molecular Genetics, University of California at Los Angeles, Los Angeles, California, United States of America; University of British Columbia, Canada

## Abstract

Most modern eukaryotes diverged from a common ancestor that contained the α-proteobacterial endosymbiont that gave rise to mitochondria. The ‘amitochondriate’ anaerobic protist parasites that have been studied to date, such as *Giardia* and *Trichomonas* harbor mitochondrion-related organelles, such as mitosomes or hydrogenosomes. Yet there is one remaining group of mitochondrion-lacking flagellates known as the Preaxostyla that could represent a primitive ‘pre-mitochondrial’ lineage of eukaryotes. To test this hypothesis, we conducted an expressed sequence tag (EST) survey on the preaxostylid flagellate *Trimastix pyriformis*, a poorly-studied free-living anaerobe. Among the ESTs we detected 19 proteins that, in other eukaryotes, typically function in mitochondria, hydrogenosomes or mitosomes, 12 of which are found exclusively within these organelles. Interestingly, one of the proteins, aconitase, functions in the tricarboxylic acid cycle typical of aerobic mitochondria, whereas others, such as pyruvate:ferredoxin oxidoreductase and [FeFe] hydrogenase, are characteristic of anaerobic hydrogenosomes. Since *Trimastix* retains genetic evidence of a mitochondriate ancestry, we can now say definitively that all known living eukaryote lineages descend from a common ancestor that had mitochondria.

## Introduction

The origin of the eukaryotic cell and mitochondria were major transitions in the evolution of life. However, the mechanisms and the temporal ordering of events underlying these transitions remain poorly understood. There are two main kinds of hypotheses regarding the sequence of events for these transitions. The first kind invokes the origin of the nucleus, cytoskeleton and endomembrane system to yield an amitochondriate eukaryote, followed later by the acquisition of the mitochondrion through endosymbiosis [1]–[3]. The second kind proposes that the mitochondrial endosymbiosis is the key innovation in eukaryogenesis, occurring simultaneously with the formation of the nucleus, or even beforehand [4], [5]. An important difference between these scenarios is that the first predicts that primitively amitochondriate eukaryotes (Archezoa) exist, or once existed but are now extinct, whereas, according to the latter hypotheses, no such organisms ever existed. Among unicellular eukaryotes there are several taxa that lack classical mitochondria (e.g. diplomonads, trichomonads, *Entamoeba,* pelobionts, *Cryptosporidium*, chytrid fungi, microsporidia, some ciliates and heterolobosea) and some of these groups were thought to actually be representatives of primitively amitochondriate Archezoa [6]. However, genes of mitochondrial origin have been identified in all of these groups indicating that they contain (or once contained) an organelle homologous to mitochondria [7]–[22]. The nuclear location of most genes encoding mitochondrial proteins is the result of genetic transfer from the endosymbiotic α-proteobacterial ancestor of the organelle. Immunological detection of proteins that function within the mitochondrion, such as those involved in iron-sulphur cluster biogenesis (IscS, IscU) or in protein import and refolding (mt-hsp70, cpn60) enabled the visualization of double-membrane bounded organelles in these “amitochondriates” [12], [15], [18], [20], [23], [24]. These organelles likely share a common evolutionary history with mitochondria implying that these organisms and presumably all modern eukaryotes diverged from an organism that contained a mitochondrion or its homolog [25].

However, there is still one key lineage of amitochondriate protists that has not been investigated: the Preaxostyla. This group is comprised of oxymonads, gut symbionts in animals, and the free-living flagellates of the genus *Trimastix* [26], [27]. The phylogenetic position of Preaxostyla has not yet been firmly established (compare [28] and [29]), but they are regarded as members of a eukaryotic “supergroup” Excavata [1], [27]. To date, the monophyly of the Excavata has not been proven, and thus it remains possible (although improbable) that Preaxostyla emerge at the base of eukaryotes, a position that would be consistent with a primitively amitochondriate status for this lineage. Double membrane bounded organelles presumed to be related to hydrogenosomes and mitochondria can be found in the cytoplasm of *Trimastix* [30]–[32]. Typically, no such organelles are observed in oxymonads [33]–[35]. Bloodgood et al. [36] reported large dense cytoplasmic bodies in the oxymonad *Pyrsonympha*, however, these authors consider them to be neither hydrogenosomes nor other derivatives of mitochondria and they may represent endosymbiotic or engulfed bacteria. Carpenter et al. [37] reported membrane-bounded, rounded, electron-dense bodies present in *Saccinobaculus doroaxostylus*, but absent in other investigated species of the genus. Some of their micrographs suggest that this body may be bounded by two membranes. We have chosen *Trimastix pyriformis*, as the representative of Preaxostyla, for an expressed sequence tag (EST) survey to search for genes of mitochondrial origin. The survey revealed 19 genes typical for mitochondria or hydrogenosomes that potentially function in a mitochondrion-related organelle in *Trimastix*.

## Results and Discussion

We constructed a cDNA library from *Trimastix pyriformis* and sequenced 9615 expressed sequence tags (ESTs) that grouped into 2686 clusters. Using our bioinformatic tool Blastcompare, we found genes that unambiguously code for proteins functioning in mitochondria and related organelles in other organisms. In addition to these genes, we also found genes coding for the typical hydrogenosomal enzymes pyruvate:ferredoxin oxidoreductase (PFO), [FeFe] hydrogenase and two [FeFe] hydrogenase maturases–hydE and hydG. The complete list of putative organellar genes is given in Table 1.

**Table 1:** Information on the putative organellar genes in *Trimastix*.

Product	Nr. of ESTs	Sequence accession numbers	N-terminal extension	Specific for mitochondria and plastids in eukaryotes	Tree fig.
**Pyruvate:ferredoxin oxidoreductase** * $ Pyruvate oxidative decarboxylation	20	EU086497	No	No	S1
**[FeFe] hydrogenase** * Hydrogen production	27	EU086507, EU086508, EU086509	No	No	2A
**hydE** Maturation of [FeFe] hydrogenase	2	EC836286.1, EC830619.1	?	?†	-
**hydG** Maturation of [FeFe] hydrogenase	3	EC839247.1, EC836834.1, EC830944.1	?	?†	-
**Aconitase** TCA cycle enzyme	11	EU086483, EU086484	No	Yes	S2
**H-protein of glycine cleavage system** central protein in GCS	1	EU086492	Yes	Yes	S7
**P1-protein of GCS** Glycine dehydrogenase (decarboxylating) subunit 1	2	EU086490	Yes	Yes	S5
**P2-protein of GCS** Glycine dehydrogenase (decarboxylating) subunit 2	5	EU086491	?	Yes	S6
**L-protein of GCS** * $ Dihydrolipoyl dehydrogenase	2	EU086501, EU086502	No	Yes	S4
**T-protein of GCS** Aminomethyltransferase	6	EU086485	Yes	Yes	S3
**Lipoyltransferase** Lipoylisation of enzymes	1	EU086495	?	Yes	S8
**Pyridine nucleotide transhydrogenase alpha** NAD and NADP interconversion	5	EC831884, EC832444, EC832298, EC837926, EC836257	?	Yes	-
**Pyridine nucleotide transhydrogenase beta** NAD and NADP interconversion	1	EU086499	?	Yes	S9
**TOM40** Protein transport	3	EU086500	?	Yes	2B
**Mitochondrial processing protease - large subunit** * Targeting sequence cleavage	2	EU086496	No	Yes	S10
**Cpn60** * Protein folding	1	EU086489	Yes	Yes	2C
**Mitochondrial carrier 1** Transport of molecules across membrane	4	EU086488	?	No	S11
**Mitochondrial carrier 2** Transport of molecules across membrane	2	EU086487	?	No	S11
**Mitochondrial carrier 3** Transport of molecules across membrane	8	EC831184, EC835350, EC835010, EC838819, EC834757, EC836477, EC839962, EC834636	?	No	-

* The 5′ end of the cDNA obtained by 5′ RACE,

$ Genomic sequence determined, ?–N-terminal sequence is incomplete,

† data are available for only two eukaryotes.

The codon usages of the genes are similar to the codon usages of other *Trimastix* genes. Preferred codons have relatively high GC content and most often contain C in the 3^rd^ position. This observation increased our confidence that the genes originated from *Trimastix* transcripts and do not represent the contamination from bacteria that are present in the culture.

**Figure 1:**
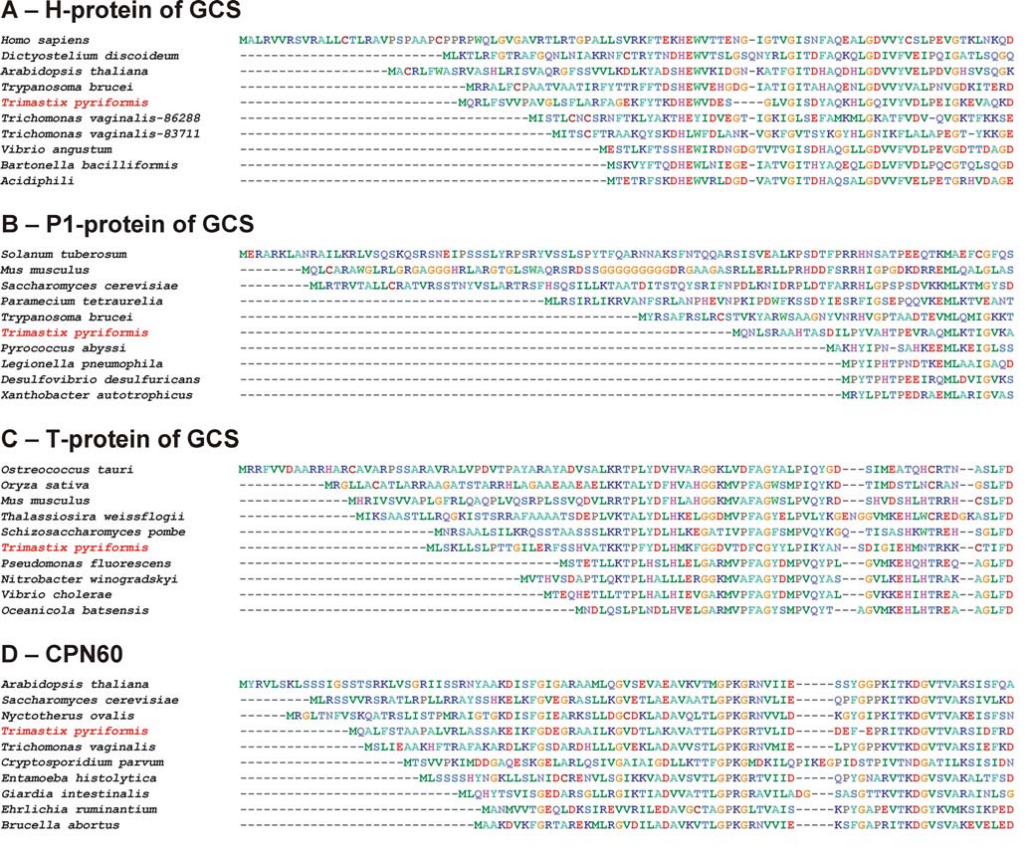
The N-terminal portion of alignments showing the extensions on *Trimastix* sequences. (A) H-, (B) P1-, (C) T-proteins of GCS and (D) cpn60.

We used rapid amplification of cDNA ends (RACE) to characterize the full-length sequences of some of these genes. For nine genes, the N-terminus of the protein sequence was identified. Four of them contained N-terminal extensions compared to bacteria. They were not recognized as mitochondrial pre-sequences by prediction software TargetP and Mitoprot, but possessed relevant hallmarks of targeting signals–rich in small, hydroxylated, hydrophobic and positively charged amino acids (Table 1, Figure 1).

The cellular localization of the gene products was not proven experimentally; however, we could tentatively infer their localization based on their phylogenetic relationship to other mitochondrial or hydrogenosomal homologs and, if present, on the putative N-terminal targeting peptides. The putative organellar proteins fell into five functional classes that are reviewed below.

### Energy metabolism

We identified key enzymes of anaerobic energy metabolism, PFO and [FeFe] hydrogenase and one protein involved in the energy metabolism of typical aerobic mitochondria, aconitase.

In the majority of aerobic eukaryotes, decarboxylation of pyruvate to form acetyl-CoA is performed by the pyruvate dehydrogenase complex (PDH), which is located in mitochondria. By contrast, in anaerobes, the non-homologous enzyme PFO typically catalyzes this reaction and, in the process, electrons are transferred to a ferredoxin. In the hydrogenosomes of chytrid fungi, pyruvate is degraded by yet another type of enzyme, pyruvate:formate lyase, and no electrons are released. PFO can be located in the cytosol (i.e. *Giardia, Entamoeba*) [38], [39] or in hydrogenosomes (i.e. *Trichomonas*) [40]. In our phylogenetic analysis, eukaryotic PFO sequences formed a single, poorly-supported clade (Figure S1), in which the *Trimastix* sequence emerged as a sister lineage to two cytosolic *Entamoeba* sequences (1.00 PP/67% BP).

**Figure 2:**
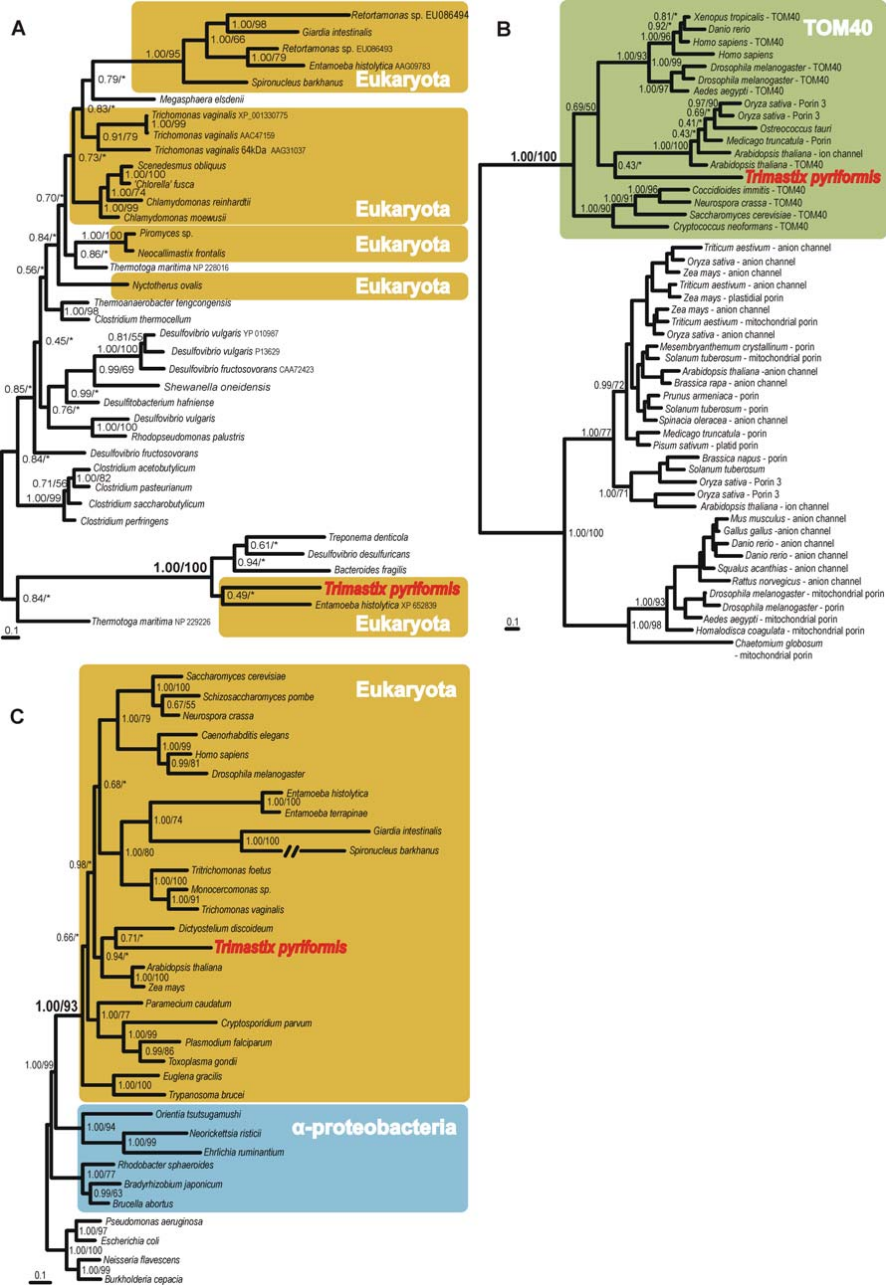
Phylogenetic trees of (A) [FeFe] hydrogenase, (B) TOM40 and (C) cpn60. Trees were constructed using MrBayes. Numbers on the branches represent statistical support expressed in Bayesian posterior probabilities/maximum likelihood bootstrap support computed with RAxML. Asterisks (*) indicate a bootstrap value of <50%. In (B) brief names of the proteins annotated according to GenBank nomenclature follow the taxon names. The statistical support for non-relevant nodes was not included for simplicity.

Hydrogenases are widely distributed among eukaryotes and prokaryotes and can be divided into three different classes: [FeFe], [NiFe] and metal-free hydrogenases [41], [42]. They can be cytosolic, membrane bound, periplasmic or organellar and catalyze the coupling of electrons with protons to form hydrogen gas or the reverse reaction. We found three distinct sequences of [FeFe] hydrogenase among the ESTs (Table 1). The sequence EU086507 was completed on the 5′ end and sequences EU086508 and EU086509 on both ends using RACE. The obtained coding sequences differed in both length (292–445 aa) and sequence (56–70 aa differences) but formed a robust clade in the tree (not shown). EU086507 corresponded to the most abundant cluster (22 ESTs) and was used for phylogenetic reconstruction (Figure 2A). The sequence branched robustly outside of most other eukaryotes in a strongly supported (1.00 PP/100% BP) group that also contained three bacterial and one *Entamoeba* sequence that weakly formed its sister branch. This gene phylogeny strongly indicated that *Trimastix* acquired its hydrogenase independently from the majority of eukaryotes. The specific relationship of both key enzymes of anaerobic metabolism, PFO and [FeFe] hydrogenase, to homologs from *Entamoeba* indicates a possible lateral gene transfer of these enzymes between *Trimastix* and *Entamoeba.* Two out of three genes required for maturation of [FeFe] hydrogenases were found among the ESTs–hydE and hydG. Although these proteins are regarded as mandatory for the production of the active [FeFe] hydrogenase enzyme in bacteria [42], they have been reported only from two eukaryotes so far, *Chlamydomonas* [43] and *Trichomonas* [44], and are absent from the draft genome sequences of *Giardia* and *Entamoeba*.

Aconitase performs stereo-specific isomerization of citrate to isocitrate and is present both in mitochondria (a part of the TCA cycle) and the cytosol, but the two types are unrelated and form distinct clades in the phylogenetic tree (Figure S2). In *Trimastix*, both types were detected but the mitochondrial one appears to be more highly expressed because it was found in 11 ESTs, in contrast to the cytosolic type, the partial sequence of which was present in only a single EST (not included in the tree).

### Amino acid metabolism

Five proteins comprising a system that decarboxylates a single amino acid were detected among the *Trimastix* ESTs. They assemble to a complete glycine cleavage system complex (GCS) that is usually located in mitochondria and performs rapid breakdown of glycine molecules to produce methyl-tetrahydrofolate and NADH. The H-protein plays a pivotal role in the GCS complex; its lipoyl group interacts with three GCS proteins: P-protein (glycine dehydrogenase [decarboxylating]), L-protein (dihydrolipoyl dehydrogenase), and T-protein (aminomethyltransferase) [45]. In bacterial systems, the P-protein is composed of two subunits that are encoded by two distinct genes. This is in contrast to eukaryotes where the P-protein is encoded as a single polypeptide. In *Trimastix*, the transcripts for the two subunits were found in two different clusters, each containing a poly A tail, indicating that they may be transcribed separately. The L-protein (dihydrolipoyl dehydrogenase) is an enzyme shared by four pathways, all of which are mitochondrial. In addition to being part of the GCS, it can function as the E3 subunit of the PDH complex, the oxoglutarate dehydrogenase complex and the branched-chain alpha-keto acid dehydrogenase complex [46]. Because the other subunits of the latter three complexes were missing among the ESTs and PFO appears to have taken the role of PDH, it is probable that the dihydrolipoyl dehydrogenase in *Trimastix* is involved exclusively in the GCS complex. Curiously, our phylogenetic analyses indicated that the GCS has a mixed evolutionary origin in *Trimastix*. While both T- and L-protein sequences were robustly embedded within the eukaryotic mitochondrial clade (Figures S3 and S4), the two P-protein subunits were robustly related to α-proteobacteria (Figures S5 and S6) and the H-protein branched weakly with bacteria, although the overall tree topology was only poorly supported (Figure S7). The N-terminus of the H-, P1- and T-proteins included extensions compared to bacterial sequences (Figure 1). It was recently reported that the *Trichomonas vaginalis* hydrogenosome also harbors some GCS subunits, however it seems that the GCS is incomplete, consisting only of H- and L-proteins, the latter being of apparent prokaryotic origin [47].

### Cofactor metabolism

Three proteins involved in metabolism of cofactors were detected–lipoyltransferase and both subunits of pyridine nucleotide transhydrogenase (PNT).

Lipoyltransferase typically performs the first step in lipoylation (covalent binding of lipoic acid) of several enzymes functioning in complexes involved in oxidative and amino acid metabolism [48], [49]. All of these enzyme complexes are present in bacteria, and those in eukaryotes are located in mitochondria and plastids. In the phylogeny of lipoyltransferase (Figure S8), the *Trimastix* homolog grouped weakly with *Dictyostelium* (0.58 PP/36% BP) as a deep branch of a moderately supported clade (1.00 PP/79% BP) that consisted mostly of eukaryotes, but included one branch of archaeal and bacterial lipoyltransferases.

PNT is an enzyme exclusively located in the inner membrane of mitochondria or the cytoplasmic membrane of bacteria and transfers hydride ion equivalents between NAD(H) and NADP(H) and, in the process, translocates protons across the inner mitochondrial/cellular membrane [50]. The enzyme is a homodimer and one monomer consists of two domains (α and β) that are expressed as two proteins in *Escherichia coli* but as a single protein in eukaryotes, e. g. *Bos*, *Eimeria* and *Entamoeba* [50]. The two domains of the *Trimastix* PNT were found in different EST clusters, each containing a poly A tail, indicating that they are transcribed separately. The sequence available for the α-subunit was too short for a reliable phylogenetic reconstruction (<<100 amino acids). In the phylogenetic tree made from β subunit sequences (Figure S9), *Trimastix* formed a branch with *Entamoeba* (1.00 PP/81% BP) at the base of an exclusively eukaryotic clade, however with relatively low support (0.99 PP/51% BP).

### Protein import and maturation

Three proteins involved in protein import and maturation were detected suggesting that the *Trimastix* organelle actively retains the ability to translocate (i.e. import) nuclear-encoded/cytoplasmically translated proteins. The proteins are: mitochondrial translocase of the outer membrane 40 (TOM40), chaperonin 60 (cpn60) and the α-subunit of the mitochondrial processing peptidase (α-MPP). The TOM complex is specific to eukaryotes and evolved early after endosymbiosis [51]. TOM40 is a β-barrel protein that forms the translocation channel in the membrane. It is an essential part of the complex and it seems to be universally distributed among eukaryotes [51], [52]. Our phylogenetic analyses strongly suggest that the *Trimastix* EST sequence is a TOM40 homolog as it is robustly (1.00 PP/100% BP) embedded in a clade of plant, animal and fungal sequences that, with a few exceptions, have been annotated as TOM40 (Figure 2B). The presence of TOM40 in the anaerobic excavate *Trimastix* further strengthens the hypothesis that this protein represents an early eukaryotic invention.

MPP is responsible for the processing of N-terminal pre-sequences after proteins have been imported into the mitochondrial matrix. MPP is active usually as a heterodimer of the paralogous alpha and beta subunits [53], the exceptions are *Trichomonas* and *Giardia*, in which only beta subunits were found [54]–[56] functioning as a homodimer [56]. The alpha-subunit participates in substrate binding and possibly product release while the catalytic activity responsible for transit peptide cleavage resides in the beta-subunit [53], [57]. All sites required for enzymatic activity [58] are present in the putative α-MPP protein we recovered from *Trimastix*. Phylogenetic analyses of MPP protein sequences robustly supported clades of the alpha and beta subunits (Figure S10) and the *Trimastix* sequence was embedded within the α-MPP clade with the *Trypanosoma* homolog as a sister branch (1.00 PP/79% BP). Eukaryote and α-proteobacterial peptidases shared a most recent common ancestor consistent with the hypothesis that MPP came into eukaryotes with the ancestor of the mitochondrion [57]. The gene duplication leading to the closely related MPP paralogs occurred very early in eukaryote history, probably before the divergence of extant eukaryotes.

Cpn60 is the mitochondrial homolog of GroEL and is involved with the refolding of proteins imported into mitochondria. This molecule is often used as a ‘mitochondrial marker’ because it unambiguously traces its ancestry to α-proteobacteria and is localized not only in mitochondria but also in the hydrogenosome of *Trichomonas* and the mitosomes of *Entamoeba* and *Giardia* [11], [15], [54]. Cpn60 from *Trimastix* branched within the eukaryote mitochondrial clade with very high statistical support (1.00 PP/93% BP), but without strong affiliation to any particular organism or group (Figure 2C). The various bioinformatic tools we employed did not recognize a mitochondrial targeting signal on the *Trimastix* cpn60 protein, however it does possess an N-terminal extension relative to bacterial homologs (Fig. 1).

### Transport of other molecules

Three members of the mitochondrial carrier family were identified among the *Trimastix* ESTs (Table 1). This diverse family of proteins facilitates the bidirectional transport of metabolites, nucleotides, amino acids, co-factors, carboxylic acids and inorganic anions, across the inner membrane of the mitochondrion. A few members were also found in the membranes of peroxisomes and plastids [59]–[61]. One member of this family, the ATP/ADP translocator, was detected in the hydrogenosomal inner membrane of *Trichomonas*, *Neocallimastix* and in the mitosomal inner membrane of *Entamoeba* [62]–[64]. The sequences of the three *Trimastix* homologs were clearly different from each other. The sequence of carrier 3 was the most divergent; moreover, its C-terminus contained a MQGP-rich repetition and showed no sequence similarity to any other eukaryote homolog. The other two carriers were included in the phylogenetic analysis of this protein family (Figure S11). Although support for the backbone tree topology was generally low, it roughly corresponded to substrate specificities. *Trimastix* carrier 1 showed a weak phylogenetic affiliation to adenine nucleotide (e.g. ATP, NAD) transporters and, in agreement with this, the sequence contained motifs characteristic for this category of carriers representing the binding site of adenine nucleotides [65]–GQ at positions 182/183 and R at position 83 (numbering according to [65]). Carrier 2 did not contain any specific binding site motifs and clustered with pyruvate and folate transporters in the tree.

### Conclusions

Among the ESTs we identified 12 proteins that are unique to mitochondrial- or plastid-derived organelles and have never been observed in other cellular compartments of eukaryotes (Table 1). Although we do not have direct evidence of organelle targeting, four of these proteins show clear N-terminal extensions. The proteins known to be specific to the outer (TOM40) or inner (PNTα, PNTβ) membrane of mitochondria are also very unlikely to function in other ‘non-mitochondrial’ membranes in *Trimastix* and the traditional function assigned to MPP makes sense only if localized in the organellar matrix.

Considering these data, there is little doubt that *Trimastix* had a mitochondriate past. Furthermore, given the presence of N-terminal extensions on mitochondrion ‘hallmark’ proteins as well as our finding of a component of the protein import system it seems likely that *Trimastix* contains anaerobic organelles homologous to mitochondria. These in turn likely correspond to the densely-staining double membrane bounded structures described in electron micrographs of these organisms [30]–[32]. As the Preaxostyla (*Trimastix* and oxymonads) were the last major candidate primitive ‘pre-mitochondrial’ eukaryote group, we can now say definitively that all known extant eukaryote lineages diverged after the mitochondrial symbiosis.

Enzymes typical for anaerobic metabolism and the characteristic enzymes of hydrogenosomes (PFO and [FeFe] hydrogenase) are relatively highly-expressed in *Trimastix* (each comprised >0.2% of the ESTs). Future localization studies are needed to establish if these enzymes are indeed active in the mitochondrion-derived organelles as in hydrogenosomes or in the cytosol, like in mitosome-containing eukaryotes. The potential presence of a TCA cycle enzyme in the organelle indicates that *Trimastix* harbors another unique version of anaerobic mitochondrion-like organelles with a unique spectrum of metabolic properties.

## Materials and Methods

### Cultures and Molecular biology

*Trimastix pyriformis* (ATCC 50935) was grown at room temperature under anaerobic/microaerophilic conditions in 1 litre tissue culture flasks (tightly sealed) in Sonneborn's *Paramecium* medium (ATCC 802 medium) pre-inoculated with *Stenotrophomonas maltophilia* as the sole food source. Cells in exponential growth were harvested by centrifugation at 1200×g for 10 minutes at 4°C. Total RNA was isolated via Tri-reagent (Sigma-Aldrich). Approximately 3mg of total RNA was sent to a commercial vendor (Agencourt Bioscience, Beverly MA, USA), to construct the cDNA library used for EST sequencing.

Both 5′ and 3′ RACE were performed from oligo-capped 1^st^ strand cDNA. PolyA+ RNA was enriched from approximately 1 mg of total RNA with the Poly(A) Purist kit (Ambion, Austin TX) and full-length 1^st^ strand cDNA was prepared with the GeneRacer Kit using SuperScript III RT (Invitrogen, Carlsbad CA). This served as template for both 5′ and 3′ RACE utilizing Taq DNA polymerase (Sigma) with either the GeneRacer 5′ or 3′ primer (GR5′/GR3′ primer) plus a gene specific primer. Often nested reactions were necessary to obtain the desired RACE products: NestedGR5′/NestedGR3′ primer plus a nested gene specific primer, plus 0.5–1.0 µl (of 50 µl) of the primary RACE PCR reaction as template. Products were cloned into TOPO T/A pCR2.1 vector (Invitrogen) and sequenced. For genomic gene sequencing, gDNA was obtained either via Tri-reagent (as a “by product” of RNA isolation) or using the PureGene DNA isolation kit (Gentra Systems, Minneapolis MN). Gene specific primers were used for PCR and products were cloned into TOPO T/A pCR2.1 vector (Invitrogen) and sequenced.

Selected clones from the cDNA library were completely sequenced using vector and gene specific primers.

### Comparative BLAST searching of the *Trimastix* clusters

Four databases were used in ‘subtractive’ BLAST searching to identify putative mitochondrial proteins in the *Trimastix* ESTs. These included mitochondrial proteome data bases from the human mitoproteome and the yeast Mitop2 database [66], [67] and ‘subtractive’ databases, created by removing proteins matching the mitochondrial proteomes from the whole predicted proteomes of these organisms. The *Trimastix* clusters were then compared to all four databases, human/yeast non-mitochondrial subtractive and human/yeast mitoproteome, using BLAST [68]. The top scoring hits for each of the subtractive and mitoproteome databases were then compared and the corresponding queries were then sorted into one of four categories: 1) Both human and yeast top hits from the mitoproteome, 2) both human and yeast top hits from the subtractive proteome, 3) human and yeast top hits from different databases, 4) or there were no significant hits in any of the databases. 128 clusters that fell into category 1 or 3 were manually inspected by comparison to the GenBank non-redundant (nr) database using BLAST and ψ-BLAST. For the most ambiguous cases preliminary trees were constructed for the cluster sequence and a selection of its homologs from the nr database. This approach narrowed the selection to 18 clusters coding for 17 unique genes.

### Codon usage analysis and removal of probable contaminants of ESTs

Using the INCA2 software (http://www.bioinfo-hr.org/en/research/inca/), the frequencies of codons (codon usages) of the putative mitochondrial/hydrogenosomal genes were compared with the frequency of codons found in 29 other *bona fide Trimastix* genes that were downloaded from the GenBank nucleotide nr database or assembled from *Trimastix* EST project.

One transcript for a putative mitochondrial carrier (EC837420) showed considerable differences in codon usage. Since the transcript was present in a single EST clone and the clone did not contain a poly-A tail, we regarded it to be a possible contaminant of the ESTs and removed it from the list of putative organellar proteins.

A transcript encoding the B14 subunit of the mitochondrial electron transport chain complex I (EU086486) also differed in codon usage from other *Trimastix* genes. This transcript was also present in a single EST, and so we regarded its occurrence in *Trimastix* as questionable. We carried out several checks to confirm its presence in *Trimastix*: PCR and nested PCR using exact match gene specific primers on gDNA and cDNA as template, 3′ and 5′ RACE using GeneRacer (Invitrogen, Carlsbad CA) and gene specific primers, and finally hybridization of a DIG-labeled probe prepared using the PCR digoxygenin (DIG) Synthesis Kit (Roche Diagnostics Corp.) to a Southern blot of restriction enzyme digested *Trimastix* gDNA. As none of these experiments showed positive results, we regard this transcript as a probable rare contaminant of the cDNA library and we removed it from the list of putative organellar proteins.

### Construction of phylogenetic trees

Orthologs of the *Trimastix* genes were downloaded from GenBank, and from the *Trichomonas vaginalis* (http://www.tigr.org/tdb/e2k1/tvg/) and *Entamoeba histolytica* (http://www.tigr.org/tdb/e2k1/eha1/) genome projects. Sequences were aligned by ClustalW implemented in BioEdit 7.0.5.3 [69] or using the ProbCons server (http://probcons.stanford.edu/) [70]. Alignments were manually refined in BioEdit 7.0.5.3 and unambiguously aligned positions were subjected to phylogenetic analyses using RAxML [71] and MrBayes 3.1.2. [72]. The PROTMIXWAG model was used in RAxML and the branching support was assessed by 100 bootstrap replicates. Two parallel runs of four chains (temp = 0.5) were run in MrBayes 3.1.2. using the JTT+Γ model with 8 discrete-rate categories. The run was considered as converged after the average standard deviation of split frequencies dropped below 0.01. The profile of tree likelihoods was inspected and the first 25% of the trees were removed from the consensus as the burn-in.

## Supporting Information

Figure S1 Phylogenetic tree of PFO. Tree was constructed by Bayesian method. Numbers at the nodes represent statistical support expressed in Bayesian posterior probabilities/maximum likelihood bootstraps computed in RaxML. * Indicates bootstrap value below 50%.
(0.58 MB TIF)

Figure S2 Phylogenetic tree of aconitase. Tree was constructed by Bayesian method. Numbers at the nodes represent statistical support expressed in Bayesian posterior probabilities/maximum likelihood bootstraps computed in RaxML. * Indicates bootstrap value below 50%.
(0.68 MB TIF)

Figure S3 Phylogenetic tree of T-protein of GCS. Tree was constructed by Bayesian method. Numbers at the nodes represent statistical support expressed in Bayesian posterior probabilities/maximum likelihood bootstraps computed in RaxML. * Indicates bootstrap value below 50%.
(0.69 MB TIF)

Figure S4 Phylogenetic tree of L-protein of GCS. Tree was constructed by Bayesian method. Numbers at the nodes represent statistical support expressed in Bayesian posterior probabilities/maximum likelihood bootstraps computed in RaxML. * Indicates bootstrap value below 50%.
(0.52 MB TIF)

Figure S5 Phylogenetic tree of P1-protein of GCS. Tree was constructed by Bayesian method. Numbers at the nodes represent statistical support expressed in Bayesian posterior probabilities/maximum likelihood bootstraps computed in RaxML. * Indicates bootstrap value below 50%.
(0.63 MB TIF)

Figure S6 Phylogenetic tree of P2-protein of GCS. Tree was constructed by Bayesian method. Numbers at the nodes represent statistical support expressed in Bayesian posterior probabilities/maximum likelihood bootstraps computed in RaxML. * Indicates bootstrap value below 50%.
(0.71 MB TIF)

Figure S7 Phylogenetic tree of H-protein of GCS. Tree was constructed by Bayesian method. Numbers at the nodes represent statistical support expressed in Bayesian posterior probabilities/maximum likelihood bootstraps computed in RaxML. * Indicates bootstrap value below 50%.
(0.56 MB TIF)

Figure S8 Phylogenetic tree of lipoyltransferase. Tree was constructed by Bayesian method. Numbers at the nodes represent statistical support expressed in Bayesian posterior probabilities/maximum likelihood bootstraps computed in RaxML. * Indicates bootstrap value below 50%.
(0.62 MB TIF)

Figure S9 Phylogenetic tree of β subunit of pyridine nucleotide transhydrogenase. Tree was constructed by Bayesian method. Numbers at the nodes represent statistical support expressed in Bayesian posterior probabilities/maximum likelihood bootstraps computed in RaxML. * Indicates bootstrap value below 50%.
(0.54 MB TIF)

Figure S10 Phylogenetic tree of mitochondrial processing peptidase. Tree was constructed by Bayesian method. Numbers at the nodes represent statistical support expressed in Bayesian posterior probabilities/maximum likelihood bootstraps computed in RaxML. * Indicates bootstrap value below 50%.
(0.48 MB TIF)

Figure S11 Phylogenetic tree of mitochondrial carrier protein family. Tree was constructed by Bayesian method. Numbers at the nodes represent statistical support expressed in Bayesian posterior probabilities/maximum likelihood bootstraps computed in RaxML. * Indicates bootstrap value below 50%.
(0.48 MB TIF)
