# Supplementary figures and images for: Genetic Evidence for a Mitochondriate Ancestry in the ‘Amitochondriate’ Flagellate Trimastix pyriformis
Source: PLoS One. 2008 Jan 2;3(1):e1383. doi: 10.1371/journal.pone.0001383 (PMC2148110; doi:10.1371/journal.pone.0001383)

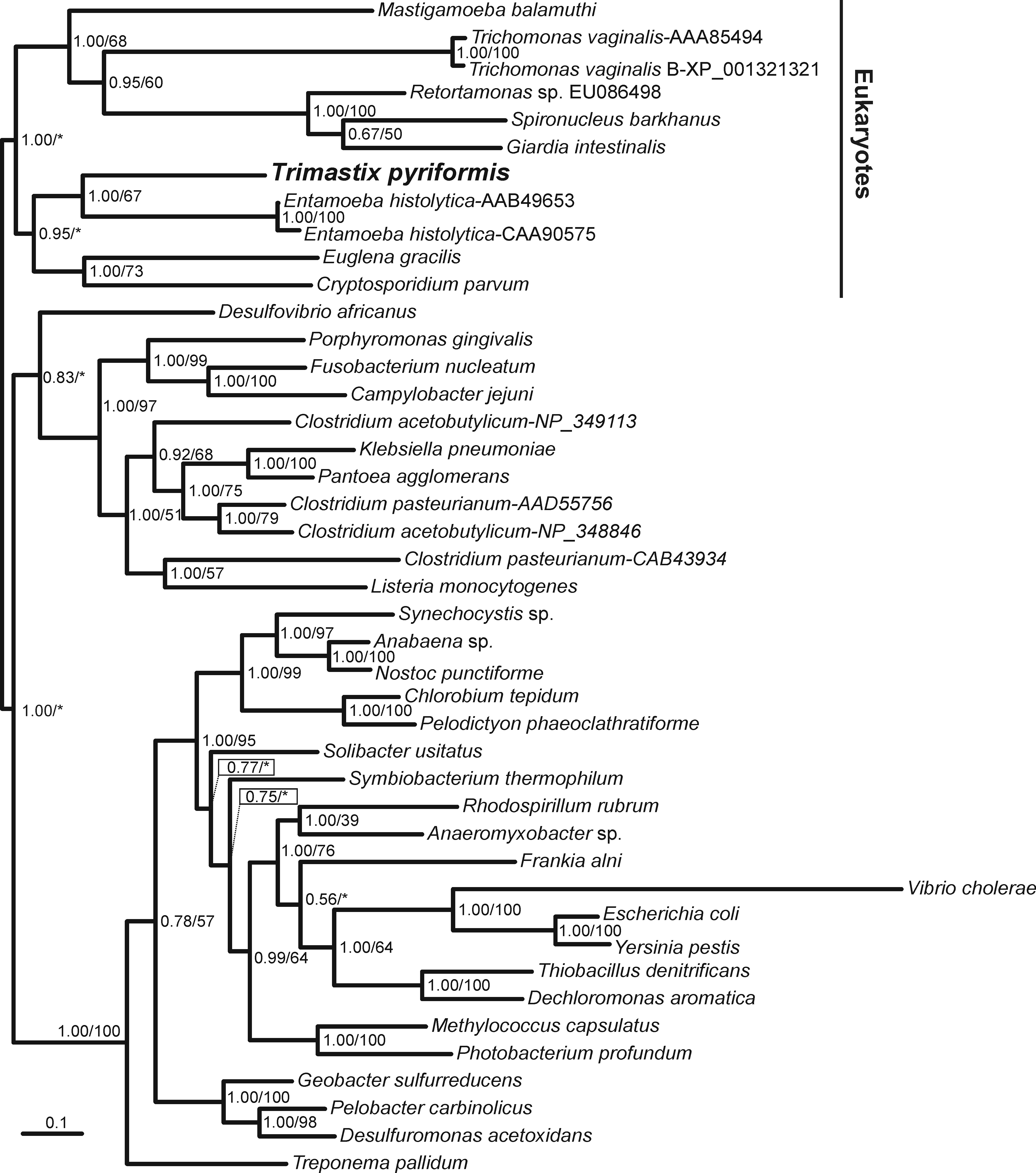

Supplement: Figure S1 — Phylogenetic tree of PFO. Tree was constructed by Bayesian method. Numbers at the nodes represent statistical support expressed in Bayesian posterior probabilities/maximum likelihood bootstraps computed in RaxML. * Indicates bootstrap value below 50%. (0.58 MB TIF) [file pone.0001383.s001.tif]

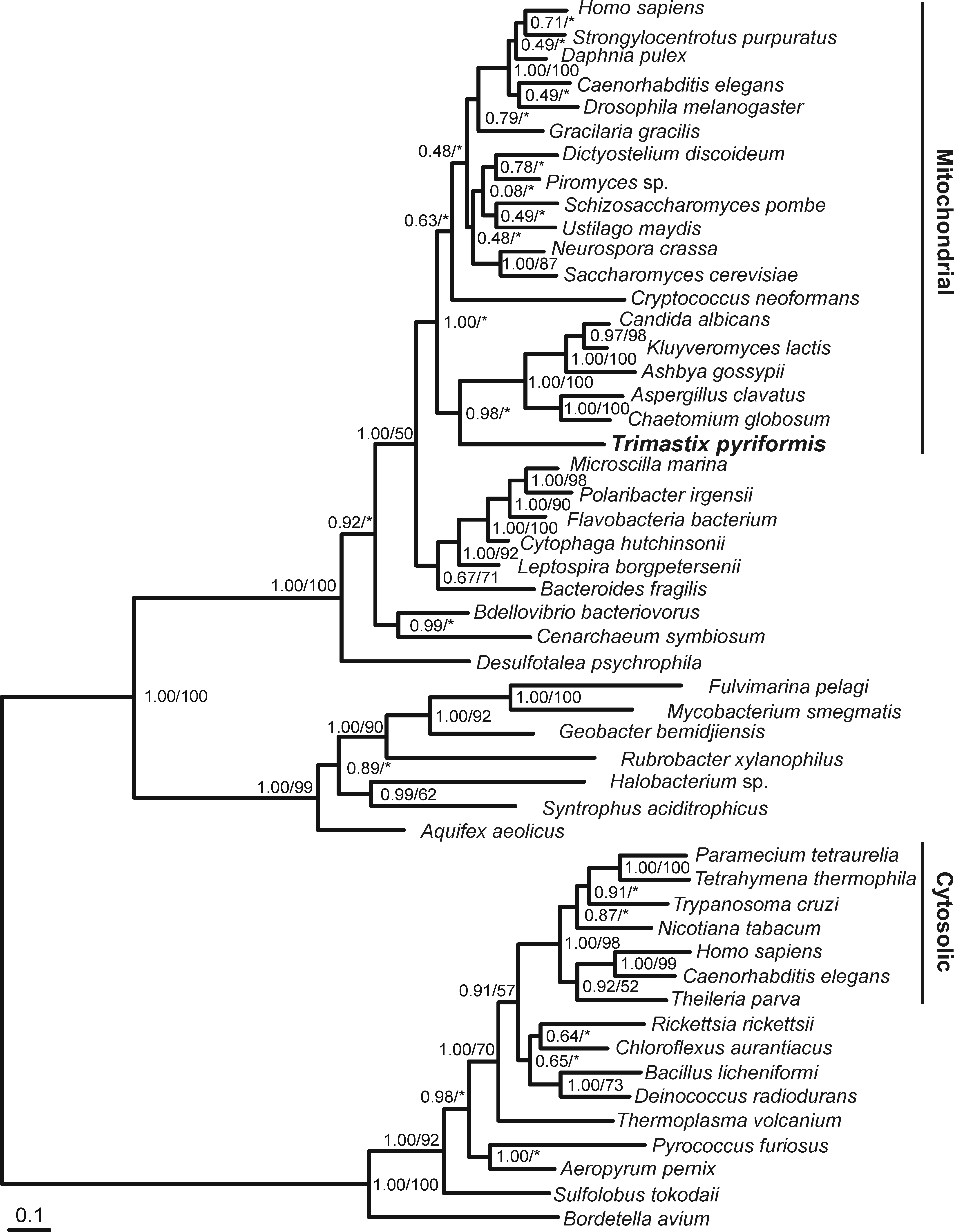

Supplement: Figure S2 — Phylogenetic tree of aconitase. Tree was constructed by Bayesian method. Numbers at the nodes represent statistical support expressed in Bayesian posterior probabilities/maximum likelihood bootstraps computed in RaxML. * Indicates bootstrap value below 50%. (0.68 MB TIF) [file pone.0001383.s002.tif]

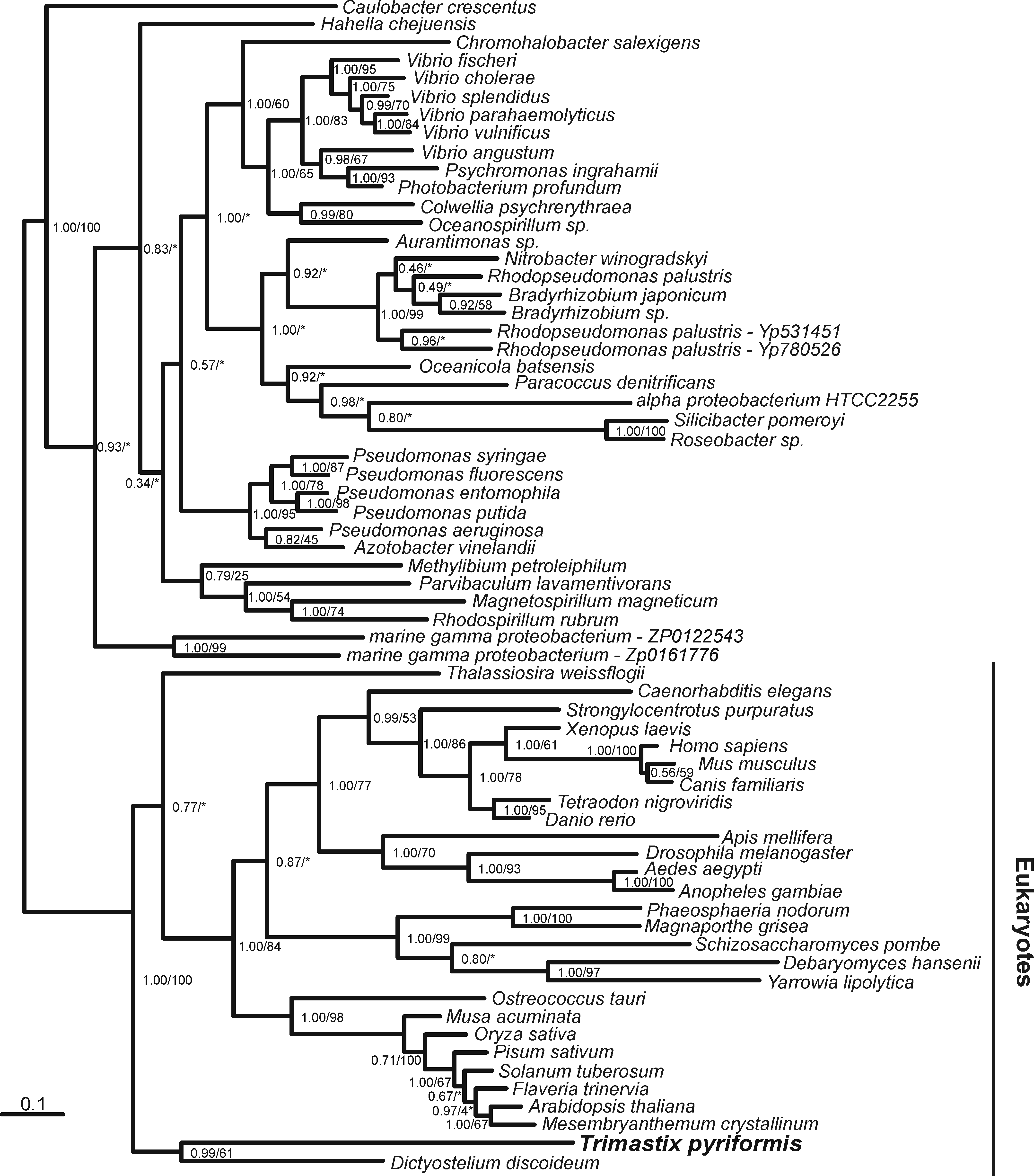

Supplement: Figure S3 — Phylogenetic tree of T-protein of GCS. Tree was constructed by Bayesian method. Numbers at the nodes represent statistical support expressed in Bayesian posterior probabilities/maximum likelihood bootstraps computed in RaxML. * Indicates bootstrap value below 50%. (0.69 MB TIF) [file pone.0001383.s003.tif]

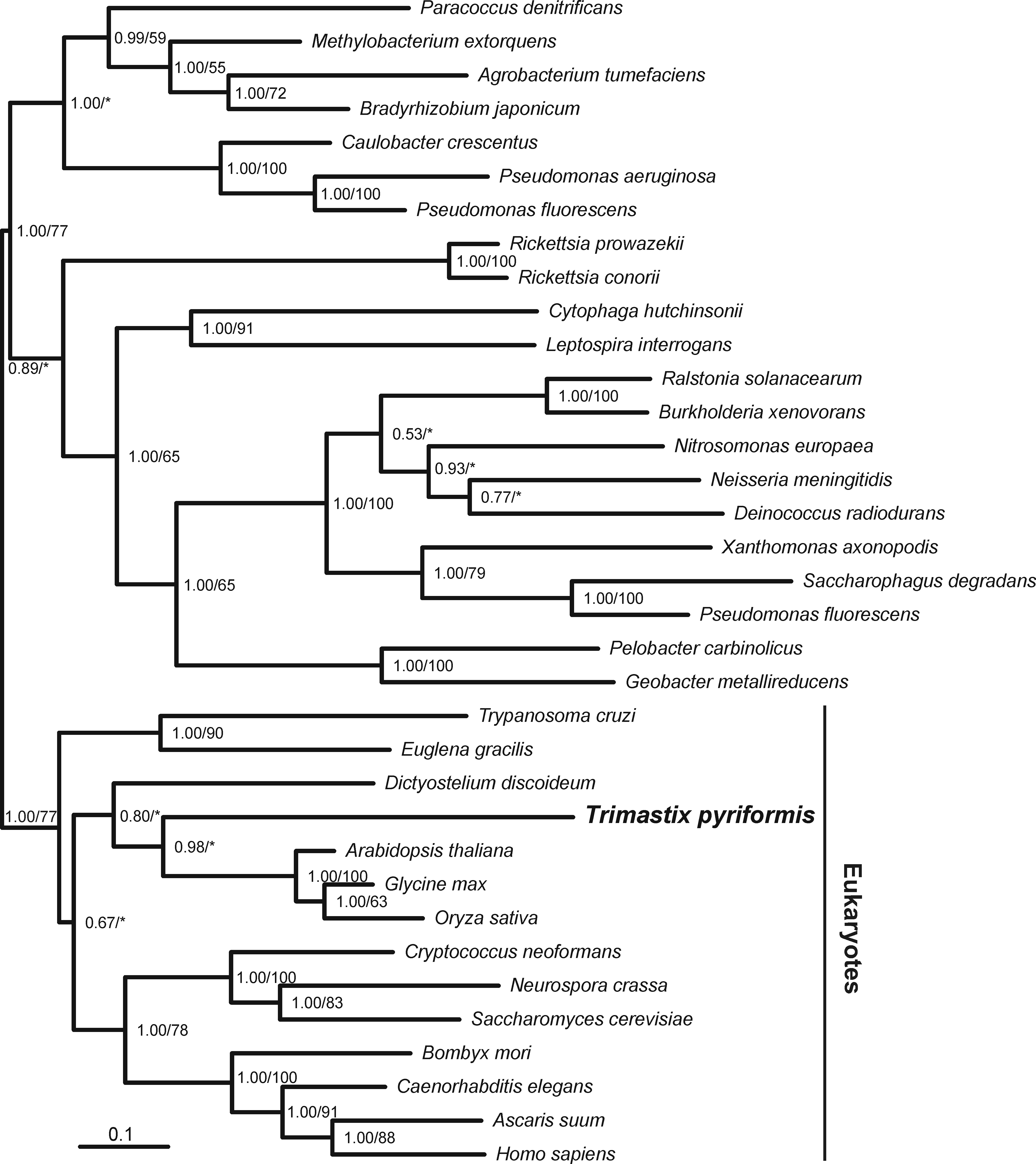

Supplement: Figure S4 — Phylogenetic tree of L-protein of GCS. Tree was constructed by Bayesian method. Numbers at the nodes represent statistical support expressed in Bayesian posterior probabilities/maximum likelihood bootstraps computed in RaxML. * Indicates bootstrap value below 50%. (0.52 MB TIF) [file pone.0001383.s004.tif]

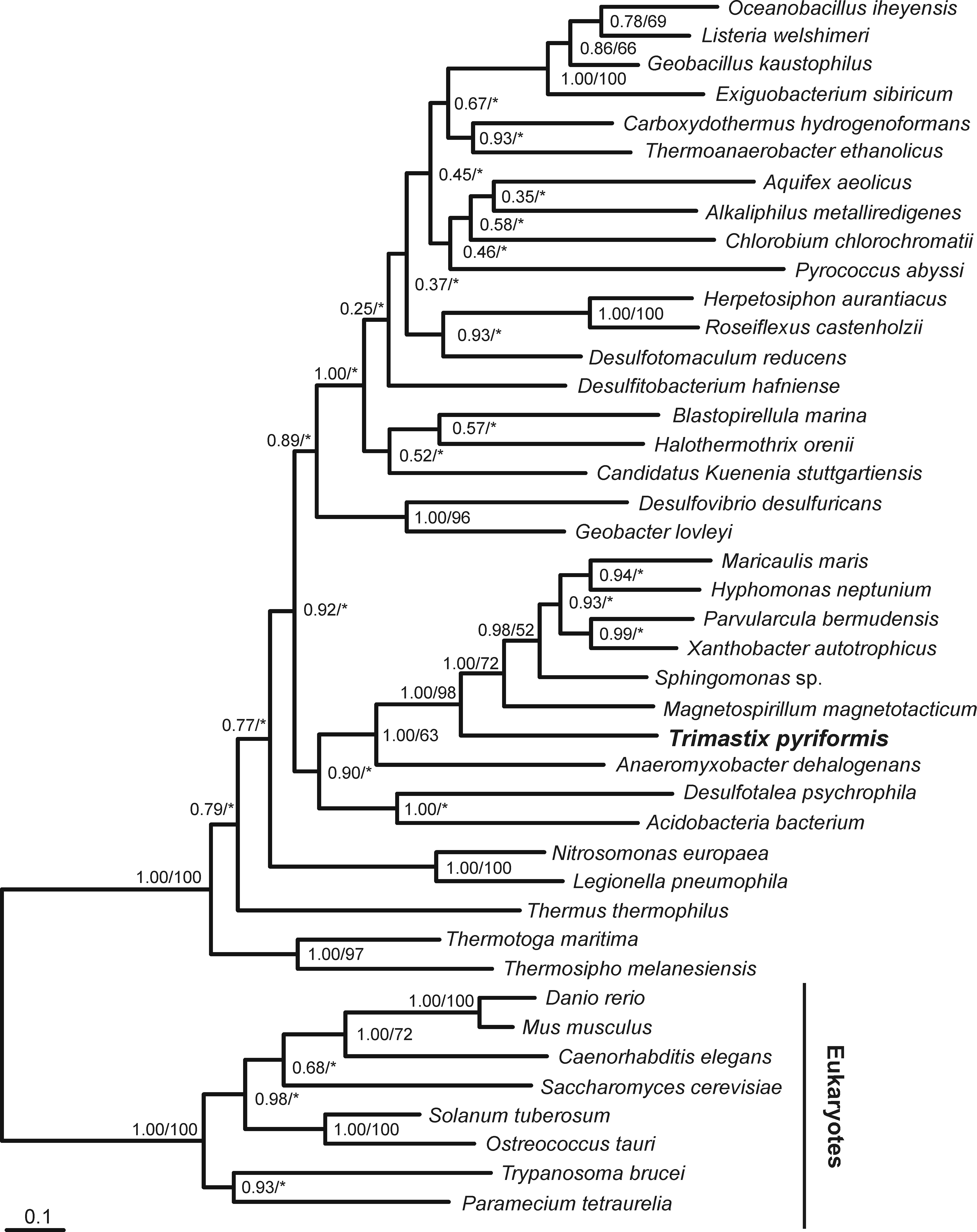

Supplement: Figure S5 — Phylogenetic tree of P1-protein of GCS. Tree was constructed by Bayesian method. Numbers at the nodes represent statistical support expressed in Bayesian posterior probabilities/maximum likelihood bootstraps computed in RaxML. * Indicates bootstrap value below 50%. (0.63 MB TIF) [file pone.0001383.s005.tif]

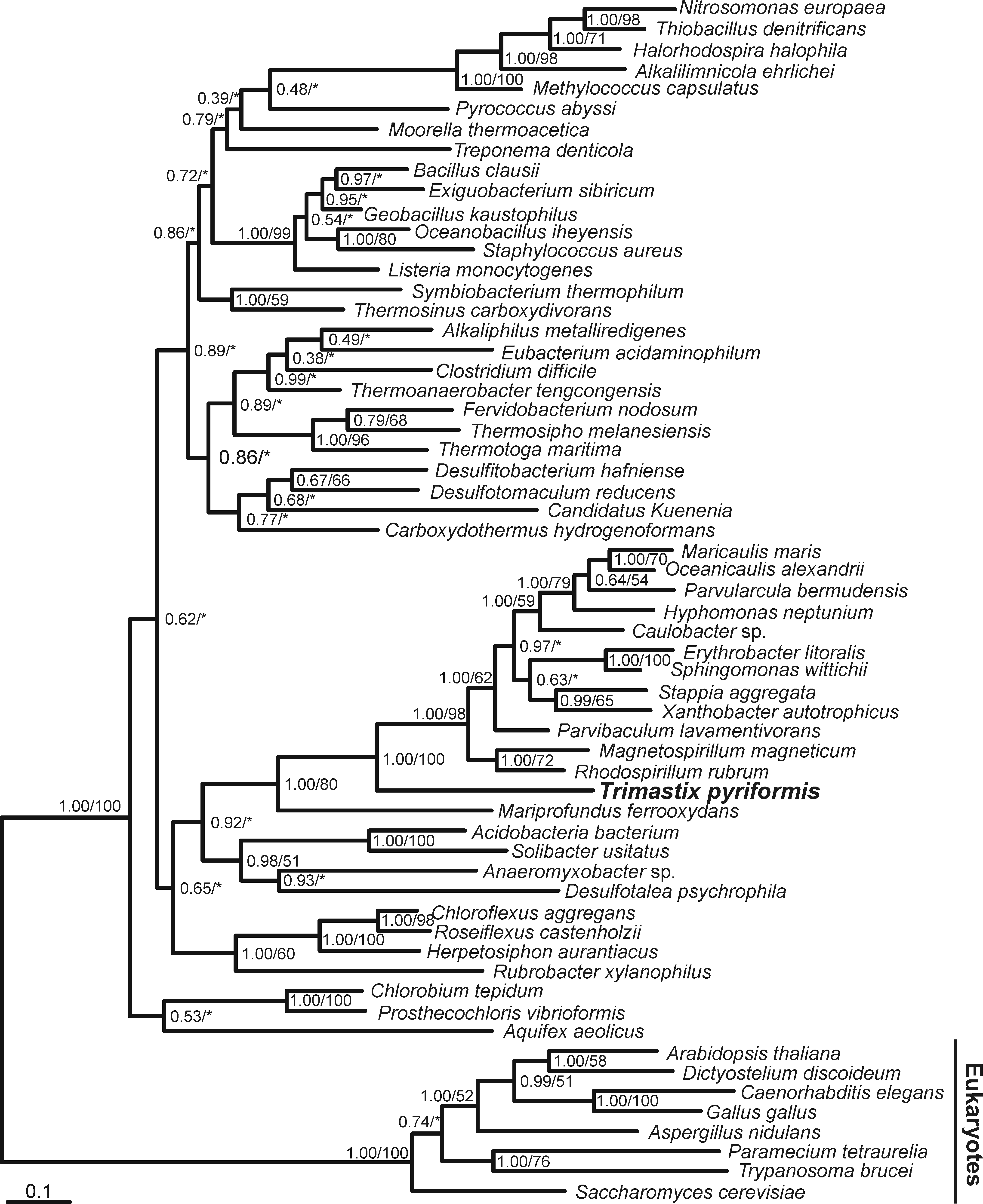

Supplement: Figure S6 — Phylogenetic tree of P2-protein of GCS. Tree was constructed by Bayesian method. Numbers at the nodes represent statistical support expressed in Bayesian posterior probabilities/maximum likelihood bootstraps computed in RaxML. * Indicates bootstrap value below 50%. (0.71 MB TIF) [file pone.0001383.s006.tif]

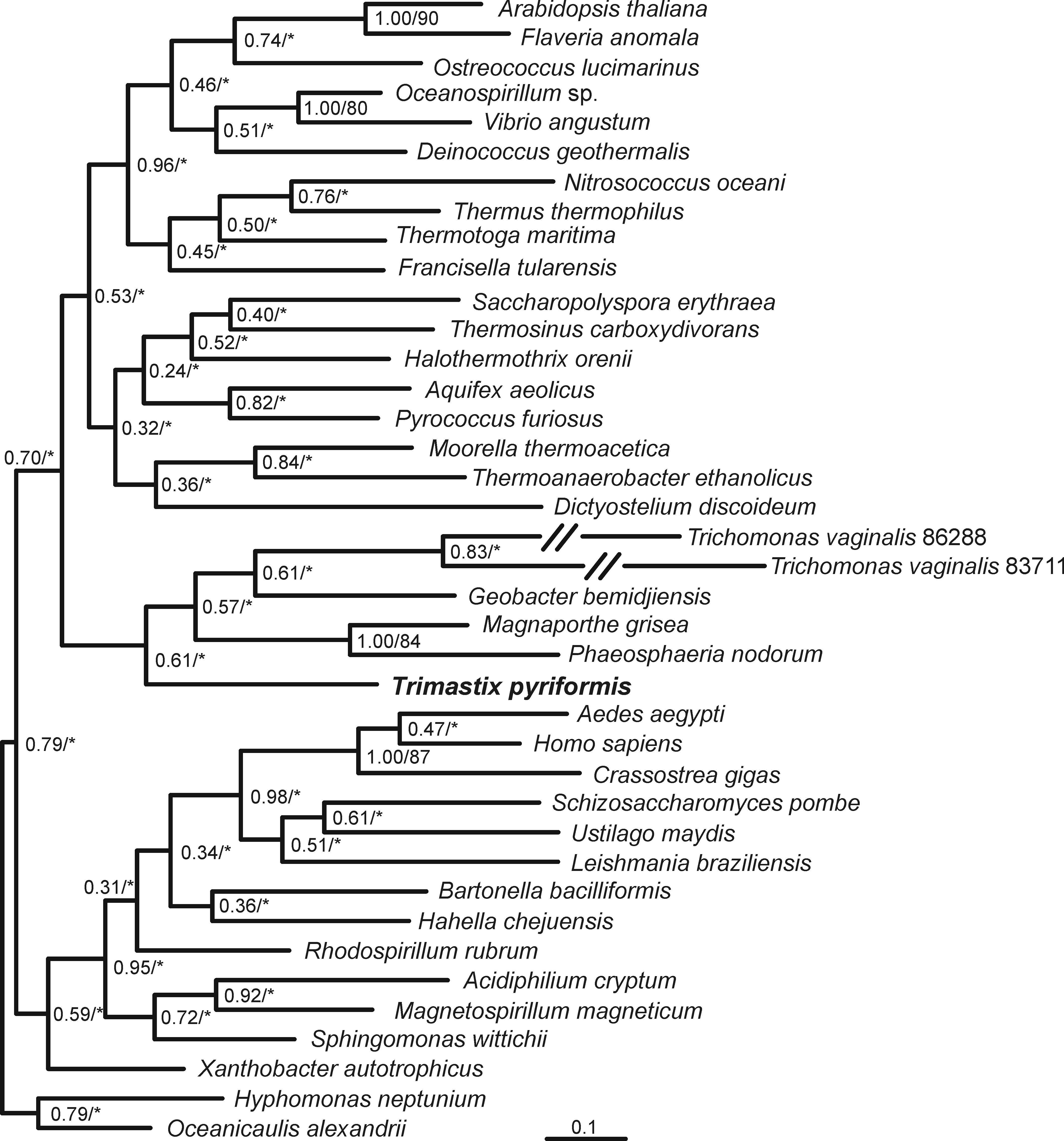

Supplement: Figure S7 — Phylogenetic tree of H-protein of GCS. Tree was constructed by Bayesian method. Numbers at the nodes represent statistical support expressed in Bayesian posterior probabilities/maximum likelihood bootstraps computed in RaxML. * Indicates bootstrap value below 50%. (0.56 MB TIF) [file pone.0001383.s007.tif]

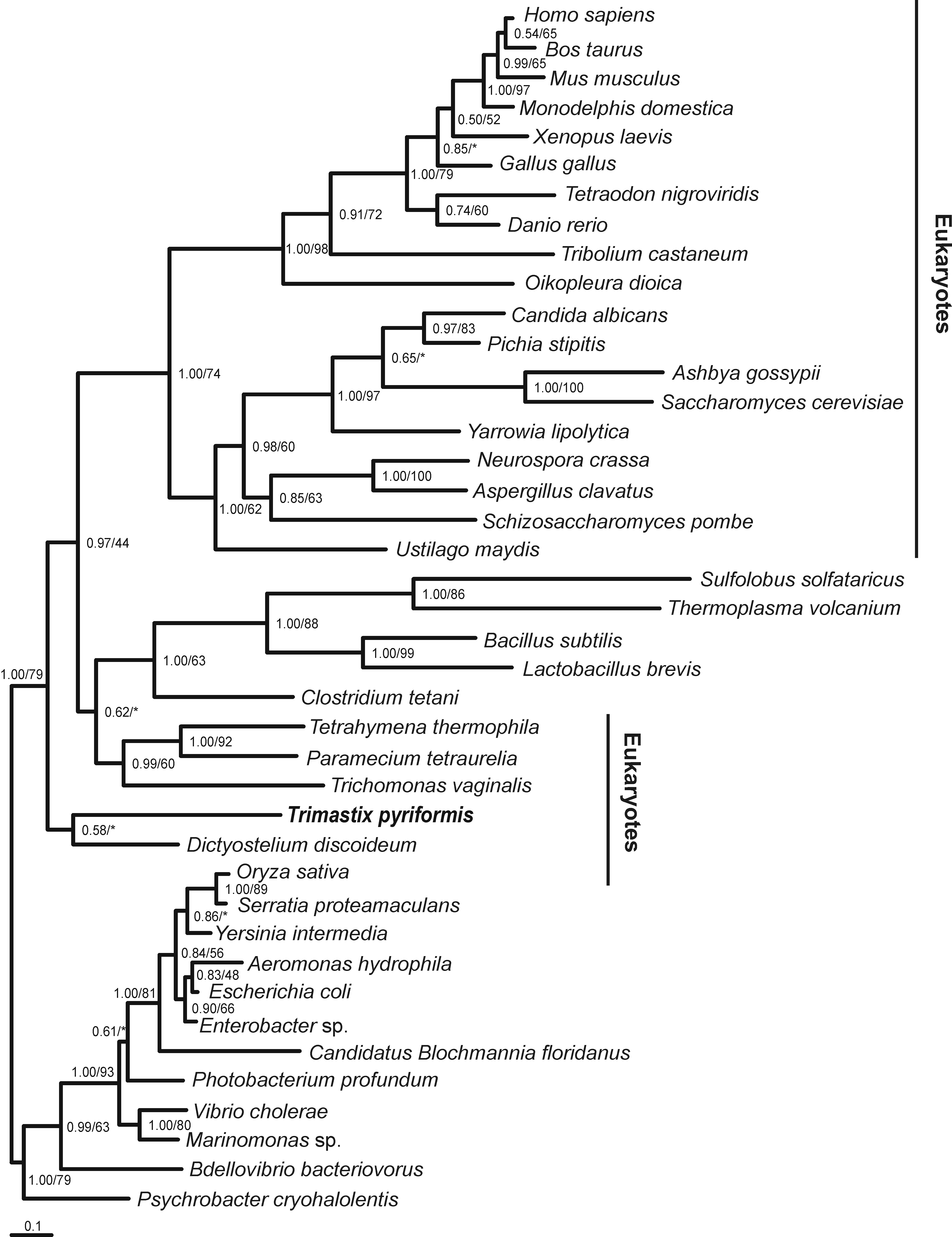

Supplement: Figure S8 — Phylogenetic tree of lipoyltransferase. Tree was constructed by Bayesian method. Numbers at the nodes represent statistical support expressed in Bayesian posterior probabilities/maximum likelihood bootstraps computed in RaxML. * Indicates bootstrap value below 50%. (0.62 MB TIF) [file pone.0001383.s008.tif]

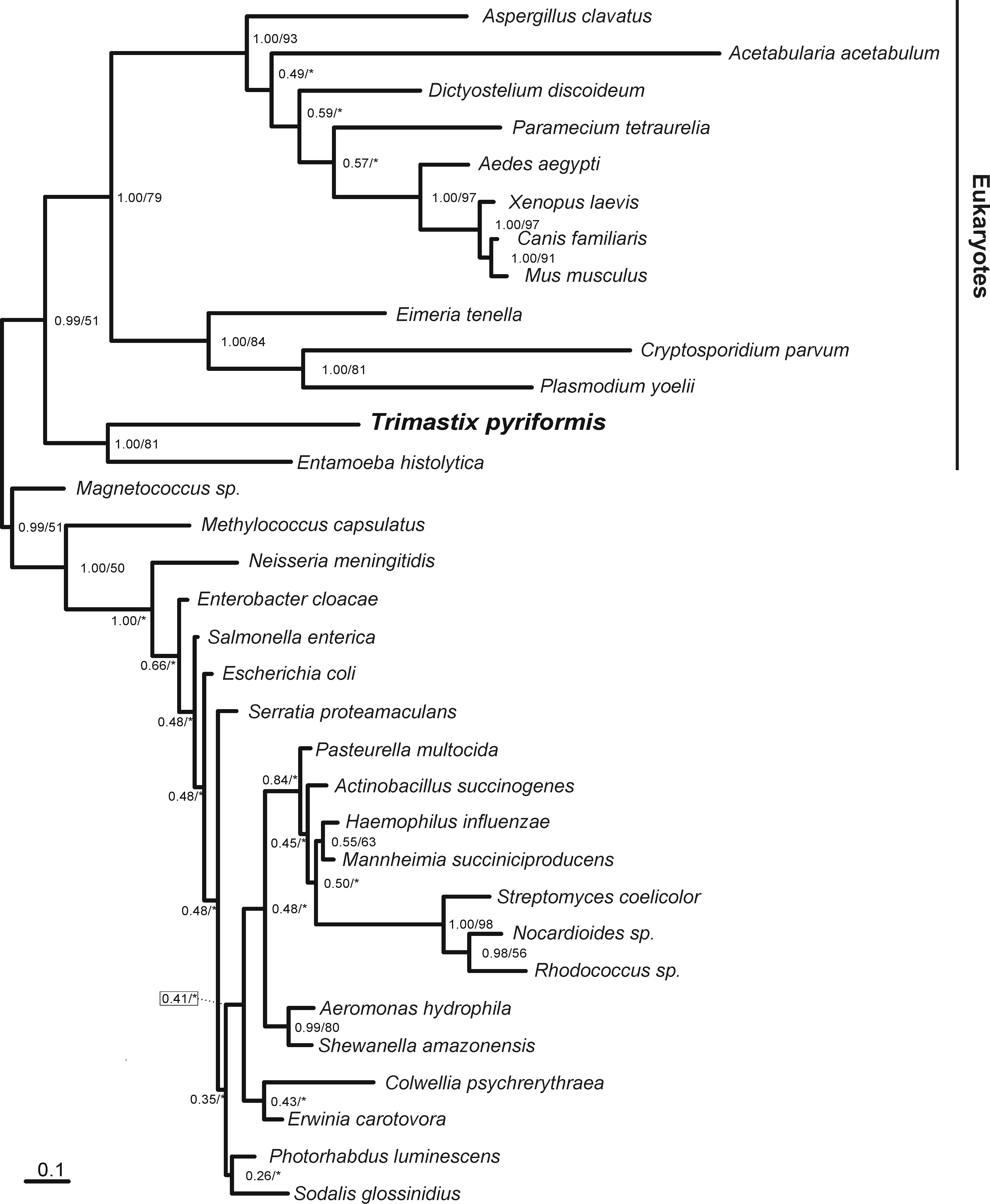

Supplement: Figure S9 — Phylogenetic tree of β subunit of pyridine nucleotide transhydrogenase. Tree was constructed by Bayesian method. Numbers at the nodes represent statistical support expressed in Bayesian posterior probabilities/maximum likelihood bootstraps computed in RaxML. * Indicates bootstrap value below 50%. (0.54 MB TIF) [file pone.0001383.s009.tif]

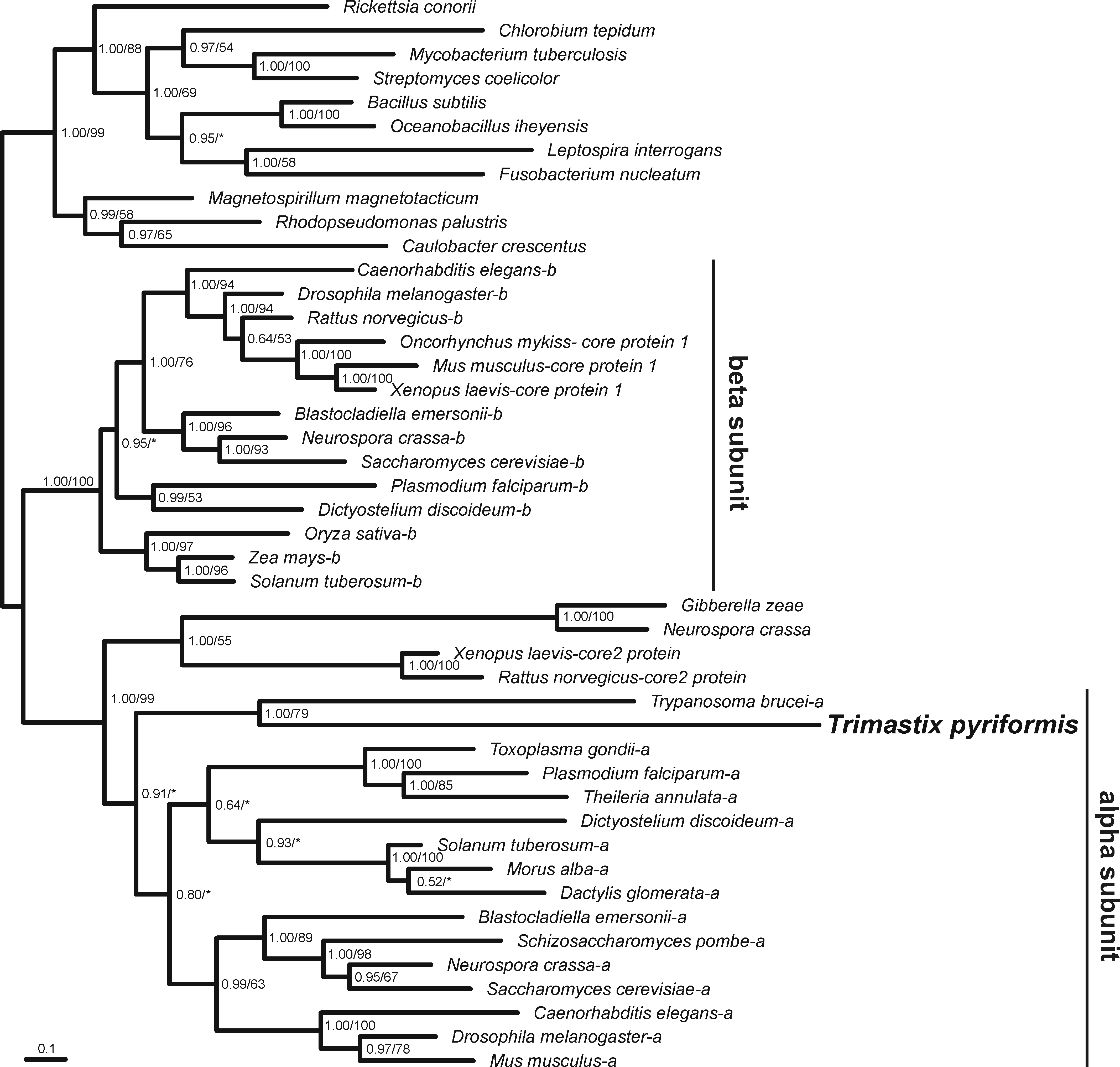

Supplement: Figure S10 — Phylogenetic tree of mitochondrial processing peptidase. Tree was constructed by Bayesian method. Numbers at the nodes represent statistical support expressed in Bayesian posterior probabilities/maximum likelihood bootstraps computed in RaxML. * Indicates bootstrap value below 50%. (0.48 MB TIF) [file pone.0001383.s010.tif]

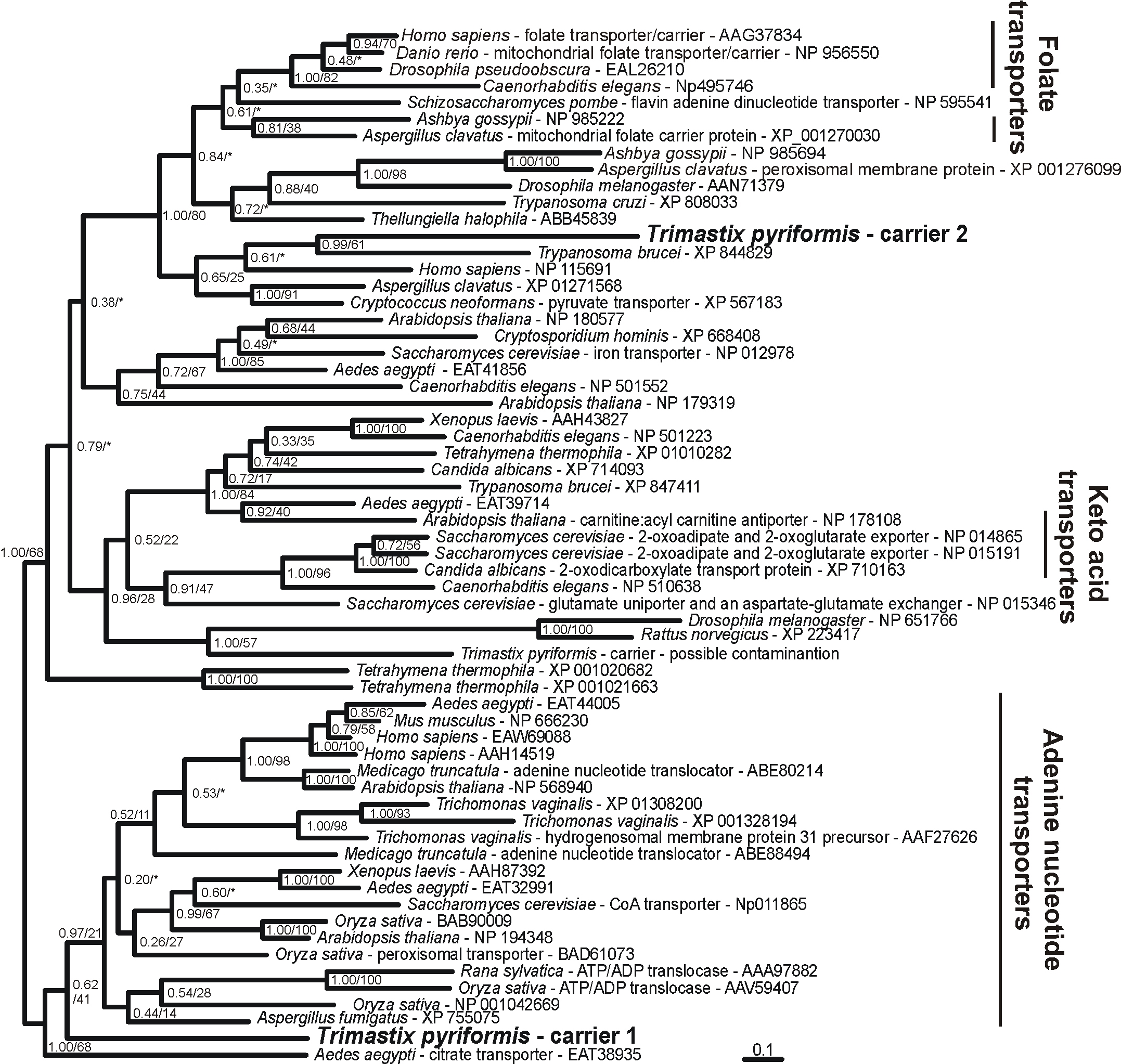

Supplement: Figure S11 — Phylogenetic tree of mitochondrial carrier protein family. Tree was constructed by Bayesian method. Numbers at the nodes represent statistical support expressed in Bayesian posterior probabilities/maximum likelihood bootstraps computed in RaxML. * Indicates bootstrap value below 50%. (0.48 MB TIF) [file pone.0001383.s011.tif]
